# Bridging the Epidemic: A Comprehensive Analysis of Prevalence and Correlates of HIV, Hepatitis C, and Syphilis, and Infection among Female Sex Workers in Guangxi Province, China

**DOI:** 10.1371/journal.pone.0115311

**Published:** 2015-02-27

**Authors:** Yi Chen, Zhiyong Shen, Jamie P. Morano, Kaveh Khoshnood, Zunyou Wu, Guanghua Lan, Qiuying Zhu, Yuejiao Zhou, Shuai Tang, Wei Liu, Jie Chen, Zhenzhu Tang

**Affiliations:** 1 Guangxi Centers for Disease Control and Prevention, Nanning, China; 2 University of South Florida, Morsani College of Medicine, Division of Infectious Disease and International Medicine, and College of Public Health, Tampa, Florida, United States of America; 3 Yale University School of Public Health, New Haven, Connecticut, United States of America; 4 National Center for AIDS/STD Control and Prevention, China Centers for Disease Control and Prevention, Beijing, China; 5 Health and Family Planning Commission of Guangxi, Zhuang Autonomous Region, Nanning, China; Johns Hopkins School of Public Health, UNITED STATES

## Abstract

**Introduction:**

Female sex workers (FSWs) are at highest risk for contracting HIV and facilitating the current heterosexual HIV epidemic in Guangxi, China, yet little is known of the impact of recent harm reduction campaigns in the province. We analyzed sentinel surveillance data collected between 2010 and 2012 in Guangxi to explore correlations between the prevalence of HIV, hepatitis C (HCV), and syphilis and risk behaviors of different categories of FSWs in Guangxi.

**Methods:**

The sentinel surveillance data for 5,1790 FSWs in all 14 prefectures and 64 city/county regions of Guangxi, China from 2010 to 2012 were collected. Differences between three categories of FSWs (grouped by venue) and disease trends (HIV, HCV, and syphilis) by year were analyzed using bivariate and multivariate logistic regression analyses as to evaluate risk factors correlated with HIV, HCV, or syphilis infection.

**Results:**

HIV and HCV prevalence remained constant across the three FSW categories; however, syphilis prevalence showed a significant increase from 5.7% to 7.3% for low-tier FSWs. Most cases with HIV, HCV, syphilis and intravenous drug use were seen in low-tier FSWs. Testing positive for HIV and syphilis were most correlated with being HCV positive (AOR 4.12 and AOR 4.36), only completing elementary school (AOR 3.71 and AOR 2.35), low tier venues (AOR 2.02 and AOR 2.00), and prior STI (AOR 1.40 and AOR 3.56), respectively. HCV infection was correlated with ever injecting drugs (AOR 60.65) and testing positive for syphilis (AOR 4.16) or HIV (AOR 3.74).

**Conclusions:**

This study highlights that low tier FSWs with lower formal education levels are the most vulnerable population at risk for acquiring and transmitting HIV, HCV, and syphilis in Guangxi, China. Condom distribution with evolution to safer sex practices are the reasons to explain the non-increasing prevalence of HIV, HCV in Guangxi for 2010–2012.

## Introduction

Heterosexual transmission has been the predominant mode of human immunodeficiency virus (HIV) acquisition in China since 2008 and currently accounts for 46.5% of the estimated total 780,000 people living with HIV and AIDS in China for 2013 [1]. Female sex workers (FSWs) are at highest risk for contracting HIV and facilitating the current heterosexual HIV epidemic and are thought to bridge transmission of HIV and sexually transmitted infections (STI) from high-risk groups to the general population [1–2]. From the early 20th century, the number of HIV infections has risen among commercial FSWs in China, many of whom are drug users who trade sex for money or drugs.

Guangxi province, with a population of 51 million [3], is representative for other regions in Asia as has witnessed an evolving HIV epidemic from transmission from needle sharing among intravenous drug users to transmission among immediate sexual contacts now currently among sexual contacts in the general population. Early HIV transmission among drug users in China originally began along heroin trafficking routes from Southeast Asia from Myanmar and Laos through northern Vietnam to China’s Guangxi Province [4–11]. This transmission pathway as well newly recombinant HIV strains demonstrating intermixing between populations using intravenous drugs and engaging in high risk heterosexual sex have since been confirmed using molecular subtyping [11–13].

For example, molecular epidemiology subtyping has confirmed the progression of the most predominant HIV-1 circulating recombinant strains among IDU’s in the Guangxi city of Beihai in 2008 (BH048/057/066) to also be transmitted to mainly heterosexual populations. This HIV-1 BH048 strain prevalence is consistent with the time point in which reported cases through heterosexual transmission exceeded that of reported cases through intravenous drug use in 2007 [14–18].Currently, of the total 36,669 HIV/AIDS cases reported in Guangxi province from 2009–2011, heterosexual transmission accounted for the vast majority of 90% of all cases [19]. The type of sex work venue is known to reflect both risk and prevalence of disease acquisition. A study conducted in Liuzhou indicated that categorization based on sex work venue was more closely related to the risk of STI than the amount of money exchanged [20]. Thus, the Chinese Centers for Disease Control and Prevention’s (China CDC) National HIV/AIDS Sentinel Surveillance Guidelines in China systematically requires that all types of entertainment venues be mapped and classified into high, middle and low tiers based on risk behaviors [21]. Though there is no official criterion for categorizing risk behaviors among FSWs specifically [22], the national surveillance venue criteria are considered the most objective for surveillance and statistical purposes. This categorizes, FSWs who work in venues that include saunas, karaoke halls (KTV), night clubs, bars, and high-ranking hotels as “high-tier” FSWs [23–24]. “Medium-tier” FSWs are classified as having work venues in hair salons, foot massage parlors, and mid-ranking hotels. “Low tier” FSWs are classified as those generally operating out of streets with poor conditions, rental houses, small eateries, rural entertainment venues, and small inns.

China’s national HIV and syphilis prevalence for FSWs is estimated at 0.04% and 3.17% respectively over 2009–2011[24]; hepatitis C (HCV) prevalence was estimated at 0.7%-0.9% between 2009 and 2012[25]. Specifically, HIV prevalence rate among low tier FSWs throughout China has been rising from 1.5% in 2010 to 2.3% in 2012, though with syphilis prevalence rate among this group constant around 7.0% in the same period [26].

Guangxi province FSWs appear to be at higher risk of HIV infection than FSWs in other Chinese provinces. A cross-sectional study of 6 cities in southern China within Guangxi, Guangdong, and Hainan provinces in 2009 revealed that FSWs from Guangxi had nearly eight times greater odds of being HIV positive as compared to other regions [27]. A previous study indicated that total HIV prevalence for low tier FSWs within Guangxi is estimated at 1.88%, while syphilis and HCV prevalence as estimated at 11.29% and 1.73% in 2011 respectively, which are higher than the national averages [28].

Despite such high disease prevalence in this risk group, little is known about the impact of recent HIV prevention and harm reduction campaigns, such as condom distribution, among FSWs in Guangxi. It was estimated in 2011 that among low tier FSWs in some places of Guangxi, consistent condom use rate was 47.2% and ever drug use at 2.4%, with 38.5% of FSWs reporting ever injecting drugs [28]. Despite evidence from a community based survey that 59.1% of female IDUs engaged in commercial sexual behavior within the past 6 months, condoms were used consistently by only 9.7% of women with regular sexual partners and 28.6% with intermittent sexual partners in the past one month [29]. As national reports indicated that FSW prevalence of HIV and syphilis declined from 2000 to 2011 throughout China[24], we sought to analyze sentinel surveillance data collected between 2010 and 2012 specifically in Guangxi to assess whether recent harm reduction interventions (condom promotion, regular HIV screening(every 6 months) and outreach intervention)introduced by the China CDC were correlated with improved HIV and syphilis epidemic trends and reductions in HIV risk behaviors among different categories of FSWs in this province.

## Methods

This longitudinal, prospective surveillance study utilizes laboratory screening data and survey results collected from April to July in 2010, 2011, and 2012 and was carried out in all 14 prefectures and 64 city/county regions in Guangxi province, China.

### Study population

The study population consisted of FSWs 4 city/county regions in Guangxi province, China, who had either currently or formerly provided commercial vaginal sex services for male clients.

As to be consistent with the National HIV/AIDS Sentinel Surveillance Guidelines in China, sex work venue [30–33], not fee charged for per sexual transaction [34–35] nor HIV prevalence [18], was used to categorize FSWs into high, middle, and low tiers. Sex work venue is directly observable and more frequently used by researchers in China and elsewhere for categorization for epidemiological purposes [36–37].It should be noted that FSW categorization by sex work venue is an epidemiological construct as to help understand differences in sexual risk behaviors and thus differing acquisition and transmission of HIV, HCV, and syphilis across FSWs subgroups in order to develop optimal, tailored, and successful harm reduction strategies.

### Questionnaire

The study utilized a standard interviewer administered, face-to-face questionnaire as part of the China CDC National HIV/AIDS Sentinel Surveillance System that included demographics, sexual risk behaviors, overall drug use, intravenous drug use, current and past STI history, and current screening test results for HIV, HCV, and syphilis. Trained study staff administered the questionnaire in either Mandarin or Cantonese, depending on the participant’s language preference.

### Recruitment

Approximately 4500 different venues were identified for surveillance and sampling sites during each year of the study. Outreach workers identified local venues to conduct patient interviews and testing through convenience sampling. Low tier and middle tier FSW tier categories were recruited with at least 50% representation from each category, and some smaller counties in Guangxi reached nearly 80–90% recruitment.

The sentinel surveillance systems collects deidentified data, no names are collected. Persons in the sentinel surveillance who volunteer for the study are assigned a unique ID code which is then linked to their questionnaire and blood sample. To ensure the anonymity of the study participants we opted for verbal consent rather than written consent. Trained study staff documented the verbal consent on their daily activity log. The choice of verbal consent was approved by the IRB of China CDC.

Verbal Informed consent was obtained for each participant for a structured interview, blood sampling and banking, and confidential HIV, HCV, and STI testing that included syphilis. A linked anonymous serial testing strategy was used for all HIV, HCV, and syphilis testing. The annual sentinel surveillance is anonymous survey that uses a unique identifier to link the questionnaire information and the resulting test results. All biological data were coded with a unique identifier and later linked with behavioral and demographic data obtained in the questionnaire.

This study was approved by the China Centers for Disease Control and Prevention Institutional Review Board.

### Laboratory methods

Each consented participant had 3–5 ml venous blood drawn for HIV, HCV, and syphilis testing using an enzyme-linked immune sorbent assay (ELISA) (Beijing Wantai Biological Pharmacy. Ltd and Zhuhai Livzon Diagnostics, Inc., China) Positive syphilis samples were confirmed with rapid plasma reagin (RPR) testing (Shanghai Kelong Bioengineering Ltd by Share Ltd, China]. HIV antibody positive samples underwent confirmatory HIV Western blot testing (Profiblot 48, TECAN company, Austria). Samples with confirmatory positive results were categorized as “surveillance positive” and appropriately included in statistical prevalence analysis. All clients with HIV, HCV and syphilis positive results received counseling and referral to the local China CDC based on city/county level of the client’s residence for follow-up testing and treatment. Seronegative clients were also advised to proceed for follow-up testing in the future as clinically indicated.

### Data analysis

Sentinel surveillance data including questionnaire and laboratory results from 2010 to 2012 were entered into Epi-Data3.1 (The EpiData Association, Odense Denmark, 2004) and then analyzed using PASW SPSS 18.0 (PASW Statistics Release18.0, Armonk, NY, USA, 2009). The Kruskal-Wallis test was utilized to determine differences between the three categories of FSWs and differences of the group sampled over the three years; the Mantel-Haenszel test was used to determine trends by year. Stepwise bivariate and multivariate logistic regression analyses were then applied to evaluate risk factor correlations with HIV, HCV, or syphilis infection. Variables with p<0.05 in the bivariate analysis were included in the final multivariate logistic regression analysis with resulting odds ratio (OR), adjusted odds ratio (AOR), and 95% confidence intervals (CIs).

## Results

### Demographics

A total of 56,293 FSWs in Guangxi were screened and represented nearly the entire estimated FSW population in the region with heaviest recruitment among middle and low tier groups. Of these screened, 51,790 FSWs were consented to participate in the 2010–2012 Guangxi China CDC HIV/AIDS Sentinel Surveillance study, which represented a much larger target population recruited than the traditional 30% of total estimated FSW recruited in Guangxi in previous years. The average age of the FSWs surveyed was 29 years with 76.4% originally from Guangxi province. Ethnic distribution reflected that of the general Guangxi population within this Zhuang Autonomous Region with 56.5% self-identifying as Han majority ethnicity and 32.8% comprised of the Zhuang minority ethnicity.

A majority of FSWs interviewed (55.2%) had completed at least junior high school (9 years of formal education). Interestingly, the majority of all FSWs were married or cohabitating with a partner (56.5%), though 39.7% reported being single or never married. Based on category of sex work venue among FSWs, 10.6% were high tier, 44.7% were middle tier, and 39.3% were low tier, though there was notably more recruitment of low tier FSWs in the subsequent years of the study due to the realization of this group being high risk for disease acquisition and transmission (Table 1,Baseline demographics and characteristics of female sex workers (FSWs), Guangxi Province, China; China National CDC HIV/AIDS Surveillance Data, 2010–2012, N = 51,790).

**Table 1:** Baseline demographics and characteristics of female sex workers (FSWs),Guangxi Province, China; China National CDC HIV/AIDS Surveillance Data, 2010–2012,N = 51,790.

FSWs	2010–2012	2010	2011	2012	
Demographic Characteristics	N = 51790	N = 16155	N = 17615	N = 18020	p-value
**Age (years)**					<0.001
Mean	29.1+/-7.7	28.0+/-7.3	28.8+/-7.6	30.3+/-7.8	
< 35	41284(79.7)	13547(83.9)	14130(80.2)	13607(75.5)	
≥36	10506(20.3)	2608(16.1)	3485(19.8)	4413(24.5)	
Category of FSW*					<0.001
High tier	5477(10.6)	1756(10.9)	2007(11.4)	1714(9.5)	
Mid tier	23170(44.7)	6591(40.8)	8761(49.7)	7818(43.4)	
Low tier	20364(39.3)	5095(31.5)	6829(38.8)	8440(46.8)	
**Birth province**					0.478
Guangxi	39584(76.4)	12345(76.4)	13512(76.7)	13727(76.2)	
Other	12073(23.3)	3765(23.3)	4051(23.0)	4257(23.6)	
**Ethnicity**					<0.001
Han	29270(56.5)	9264(57.3)	9923(56.3)	10083(56.0)	
Zhuang	16995(32.8)	5268(32.6)	5943(33.7)	5784(32.0)	
Other	4774(9.2)	1404(8.7)	1464(8.3)	1906(10.6)	
**Marital status**					<0.001
Single	20581(39.7)	7258(44.9)	7288(41.4)	6035(33.5)	
Married/Couple	29285(56.5)	8433(52.2)	9776(55.5)	11076(61.5)	
Divorced/ Widowed	1825(3.5)	439(2.7)	498(2.8)	888(4.9)	
**Education level**					<0.001
≥ High school	5919(11.4)	2042(12.6)	2146(12.2)	1731(9.6)	
Junior High school	28611(55.2)	9125(56.5)	9764(55.4)	9722(54.0)	
≤Elementary school	17110(33.0)	4937(30.6)	5634(32.0)	6539(36.3)	
**Ever used drugs**	1271(2.5)	478(3.0)	486(2.8)	307(1.7)	<0.001
**Ever injected drugs**	196(0.4)	68(0.4)	71(0.4)	57(0.3)	0.233

Note: FSW = Female Sex Worker

*Some missing records in the dataset for tiers classification, so could not make 3 tiers to be 100%.

### HIV, HCV, and Syphilis Screening

Low tier FSWs consistently demonstrated the highest prevalence of HIV, HCV, and syphilis for all three years as compared to high tier and middle tier FSWs (p<0.001), with the three-year average prevalence of HIV, HCV, and syphilis at 1.9%, 1.3%, and 10.5%, respectively (Table 2, HIV, hepatitis C, and syphilis testing results for female sex workers (FSWs) from Guangxi Province, China; China National CDC HIV/AIDS Surveillance Data, 2010–2012,N = 51,790). High tier FSWs likewise demonstrated the lowest three-year prevalence of HIV, HCV, and syphilis at 0.5%, 0.6%, and 3.2%, respectively. Overall HIV and HCV prevalence remained constant over the three years within the three FSW categories, although the overall syphilis trend showed a significant increase from 5.7% to as high as 7.3%% among total participants within the three years. For HCV positive FSWs, 15.6% (80/514) self-identified as ever injecting drugs, while for HCV negative, only 0.2% (115/51,171) (p<0.001).

**Table 2:** HIV, hepatitis C, and syphilis testing results for female sex workers (FSWs) from Guangxi Province, China; China National CDC HIV/AIDS Surveillance Data, 2010–2012,N = 51,790.

FSWs	2010–2012(N = 51,790)			2010(N = 16,155)			2011(N = 17,615)			2012(N = 18,020)			
Disease	Test No.	Pos. No.	Prev (%)	Test No.	Pos. No.	Prev (%)	Test No.	Pos. No.	Prev (%)	Test No.	Pos. No.	Prev (%)	p-value
**HIV**													
Overall	51790	536	1.0	16138	148	0.9	17615	189	1.1	18020	199	1.1	0.498
High tier	5477	25	0.5	1756	11	0.6	2007	7	0.4	1714	7	0.4	0.338
Mid tier	23170	119	0.5	6578	31	0.5	8761	51	0.6	7818	37	0.5	0.965
Low tier	20364	381	1.9	5091	96	1.9	6829	130	1.9	8440	155	1.8	0.812
			p <.001			p <.001			p <.001			p <.001	
**HCV**													
Overall	51790	516	1.0	16138	152	0.9	17566	169	1.0	18020	195	1.1	0.37
High tier	5477	32	0.6	1756	10	0.6	2004	12	0.6	1714	10	0.6	0.956
Mid tier	23170	191	0.8	6578	62	0.9	8730	60	0.7	7818	69	0.9	0.765
Low tier	20364	274	1.3	5091	61	1.2	6814	97	1.4	8440	116	1.4	0.451
			p<.001			p = 0.062			p<.001			p<.001	
**Syphilis**													
Overall	51790	3472	6.7	16138	912	5.7	17566	1274	7.3	18020	1286	7.1	<0.001
High tier	5477	174	3.2	1756	46	2.6	2004	69	3.4	1714	59	3.4	0.165
Mid tier	23170	1048	4.5	6578	290	4.4	8730	432	5.0	7818	326	4.2	0.417
Low tier	20364	2135	10.5	5091	466	9.2	6814	770	11.3	8440	899	10.7	0.02
			p <.001			p<.001			p <.001			p <.001	

Note: FSW = Female Sex Worker; Prev = Prevalence

### Condom and Drug Use Analysis

Consistent condom use increased significantly among all three FSW categories from 62.6% in 2010 to 76.6% in 2012 (p<0.001), with high tier FSWs demonstrating the highest percentage of condom use in the last month of 2012 at 87.8%. The low tier FSWs had the lowest baseline condom use at 56.3% in 2010, and this increased significantly by 2012 to 69.2%. Overall drug use (combined oral and injection) significantly decreased for all FSW categories over the three-year period from 3.0% to 1.7% (p<0.001), with the middle tier and low tier categories showing the most significant decrease in overall (ever) drug use from 3.1% to 1.7% and 1.9% to 0.9% respectively (p<0.001). Interestingly, the high-tier FSW category demonstrated the highest baseline prevalence of overall drug use at 6.0% in 2010 with no significant change over the surveillance period (p = 0.374) (Table 3, Behaviors of condom use and ever drug use among 3 categories of female sex workers (FSWs), Guangxi province, China; China CDC HIV/AIDS National Surveillance Data, 2010–2012, N = 51,790).

**Table 3:** Behaviors of condom use and ever drug use among 3 categories of female sex workers (FSWs), Guangxi province, China; China CDC HIV/AIDS National Surveillance Data, 2010–2012,N = 51,790.

FSWs	TOTAL 2010–2012	2010	2011	2012	p-value*
Behavior	% (n/N)	% (n/N)	% (n/N)	% (n/N)	% (n/N)
**Consistent condom use, in last month**					
Overall	65.0 (33662/51790)	59.8 (9661/16155)	58.8(10355/17615)	75.7(13646/18020)	<0.001
High tier	71.2 (3902/5477)	66.9 (1174/1756)	61.7 (1239/2007)	86.9(1489/1714)	<0.001
Middle tier	69.4 (16087/23170)	64.1 (4223/6591)	62.9 (5512/8761)	81.2 (6352/7818)	<0.001
Low tier	59.8 (12172/20364)	54.9 (2799/5095)	52.7 (3596/6829)	68.4 (5777/8440)	<0.001
p-value*	p<0.001	p<0.001	p<0.001	p<0.001	
**Ever used drugs (Oral or Injected)**					
Overall	2.5 (1271/51790)	3.0 (478/16155)	2.8 (486/17615)	1.7 (307/18020)	<0.001
High tier	6.0 (328/5477)	6.1 (107/1756)	6.4 (129/2007)	5.4 92/1714)	0.374
Middle tier	2.5 (575/23170)	3.2 (212/6591)	2.6 (228/8761)	1.7 (135/7818)	<0.001
Low tier	1.5 (307/20364)	1.9 (98/5095)	1.9 (129/6829)	0.9 (80/8440)	<0.001
p-value*	p<0.001	p<0.001	p<0.001	p<0.001	
**Ever injected drugs**					
Overall	15.4 (196/1271)	14.2 (68/478)	14.6 (71/486)	18.6 (57/307)	0.050
High tier	1.5 (5/328)	0.9 (1/107)	2.3 (3/129)	1.1 (1/92)	0.981
Middle tier	15.8 (91/575)	17.0 (36/212)	11.8 (27/228)	20.7 (28/135)	0.086
Low tier	30.3(93/307)	24.5(24/98)	31.8(41/129)	35.0(28/80)	0.142
p-value*	p<0.001	p<0.001	p<0.001	p<0.001	

Note: FSW = Female Sex Worker;

*p < 0.001 difference between high, middle, and low tier groups comparison for all categories in 2010, 2011, 2012.

In terms of ever injecting drugs among those who ever used drugs, low tier FSWs demonstrated the highest significant intravenous drug use history (p<0.001) with a three-year prevalence average of 30.3%. High tier FSWs reported the lowest prevalence of injection drug use history (p<0.001) with a three-year average of 1.5%. Middle tier FSW’s averaged 15.8% injection drug use prevalence over the three year period. Trends in injection drug use were constant between all three years.

### Multivariate Models for HIV, syphilis, and HCV infection

In the adjusted multivariate model, HIV positivity on screening was correlated with being HCV positive (AOR 4.12; CI 2.70–6.27), only completing elementary school education (AOR 3.71; CI 2.14–6.43) or junior high school (AOR 2.08; CI 1.20–3.60), syphilis co-infection (AOR 2.56; CI 2.06–3.20), ever injecting drugs (AOR 2.10; CI 1.04–4.23), low tier venues (AOR 2.02; CI 1.29–3.18), being divorced or widowed (AOR 1.82;CI 1.24–2.67), being greater or equal to age 35 (AOR 1.65;CI 1.34–2.05), inconsistent condom use (AOR 1.48; CI 1.23–1.78), and prior STI in last 12 months (AOR 1.40; CI 1.02–1.94) (Table 4,Demographic and risk factors correlations of HIV or syphilis infection among female sex workers (FSW), Guangxi province, China, China National CDC HIV/AIDS Surveillance Data, 2010–2012;Table 5, Multivariate models of correlates of the outcomes of testing positive for HIV, HCV, and syphilis among female sex workers (FSWs) in Guangxi Province, China, China National CDC HIV/AIDS Surveillance Data, 2010–2012, N = 51,790.).

**Table 4:** Demographic and risk factors correlations of HIV or syphilis infection among female sex workers (FSW), Guangxi province, China, China National CDC HIV/AIDS Surveillance Data, 2010–2012.

FSWs	HIV*				Syphilis^¶^				HCV#			
Characteristic	univariate(P Value)	95%CI for OR	Multivariate(P value)	95%CI for AOR	univariate(P Value)	95%CI for OR	Multivariate(P value)	95%CI for AOR	univariate(P Value)	95%CI for OR	Multivariate(P value)	95%CI for AOR
**Category**												
High tier		Ref.		Ref.		Ref.		Ref.		Ref.		Ref.
Mid tier	0.589	1.126 (0.731–1.736)	0.630	0.892(0.562–1.418)	0.000	1.446(1.228–1.702)	0.107	1.148(0.971–1.359)	0.069	1.416(0.973–2.062)	0.454	1.157(0.790–1.696)
Low tier	0.000	4.159(2.772–6.240)	0.002	2.024(1.290–3.176)	0.000	3.571(3.051–4.180)	0.000	2.002(1.694–2.366)	0.000	2.322(1.608–3.352)	0.024	1.558(1.061–2.288)
**Age**												
<35		Ref.		Ref.		Ref.		Ref.		Ref.		Ref.
> = 35	0.000	3.364(2.834–3.992)	0.000	1.653(1.335–2.047)	0.000	2.873(2.675–3.087)	0.000	1.707(1.564–1.864)	0.000	1.470(1.209–1.787)	0.772	0.965(0.760–1.225)
**Marital Status**												
Married/Cohabiting		Ref.		Ref.		Ref.		Ref.		Ref.		Ref.
Not married/	0.000	2.048(1.668–2.514)	0.919	1.013(0.787–1.304)	0.000	2.235(2.060–2.424)	0.000	1.275(1.157–1.405)	0.039	1.217(1.010–1.467)	0.748	0.964(0.773–1.203)
Divorced/ Widowed	0.000	4.936(3.565–6.834)	0.002	1.822(1.243–2.671)	0.000	2.970(2.522–3.497)	0.003	1.326(1.104–1.594)	0.000	2.269(1.574–3.272)	0.126	1.391(0.911–2.123)
**Region of Birth**												
Guangxi		Ref.				Ref.		Ref.		Ref.		
Other	0.360	1.096(0.901–1.334)	-	-	0.000	1.309(1.212–1.414)	0.012	1.111(1.023–1.206)	0.055	1.211(0.996–1.472)	_	_
**Education Level**												
High school (12 years education) or above		Ref.		Ref.		Ref.		Ref.		Ref.		
Junior school(9 years education)	0.004	2.040(1.255–3.316)	0.009	2.077(1.199–3.599)	0.000	1.792(1.526–2.105)	0.000	1.571(1.324–1.866)	0.279	1.183(0.872–1.605)	_	_
Elementary school (6 years education)or less	0.000	6.587(4.096–10.592)	0.000	3.708(2.138–6.432)	0.000	4.063(3.463–4.766)	0.000	2.349(1.973–2.796)	0.088	1.316(0.960–1.806)	_	_
**Years living in current city for providing commercial sex service**												
0.5 year or less		Ref.		Ref.		Ref.		Ref.		Ref.		Ref.
0.5 to 1 years	0.056	0.774(0.595–1.007)	0.090	0.788(0.598–1.038)	0.694	0.980(0.884–1.086)	0.098	0.912(0.818–1.017)	0.308	1.143(0.884–1.479)	0.217	1.185(0.905–1.552)
1 year or more	0.002	1.345(1.113–1.625)	0.640	1.051(0.854–1.293)	0.000	1.551(1.434–1.677)	0.000	1.191(1.093–1.298)	0.000	1.657(1.355–2.027)	0.014	1.320(1.057–1.648)
**Consistent Condom use in recent month**												
consistent use		Ref.		Ref.		Ref.		Ref.		Ref.		
Not consistent use	0.000	2.131(1.795–2.531)	0.000	1.478(1.230–1.775)	0.000	1.449(1.350–1.556)	0.020	1.096(1.014–1.183)	0.132	1.151(0.958–1.382)	_	_
**Ever inject drug**												
No		Ref.		Ref.		Ref.		Ref.		Ref.		Ref.
Yes	0.000	7.532(4.344–13.061)	0.039	2.095(1.038–4.229)	0.000	3.974(2.828–5.585)	0.179	1.332(0.877–2.024)	0.000	81.837(60.591–110.532)	0.000	60.654(43.424–84.721)
**Diagnosed with an STI in the last 12 months**												
No		Ref.		Ref.		Ref.		Ref.		Ref.		Ref.
Yes	0.000	2.357(1.746–3.182)	0.041	1.402(1.015–1.939)	0.000	4.033(3.608–4.507)	0.000	3.558(3.150–4.018)	0.000	2.310(1.701–3.137)	0.334	1.189(0.837–1.690)
**Syphilis(for HIV)/ HIV (for Syphilis and HCV)**												
Negative		Ref.		Ref.		Ref.		Ref.		Ref.		Ref.
Positive	0.000	4.965(4.070–6.057)	0.000	2.564(2.057–3.195)	0.000	4.965(4.070–6.057)	0.000	2.609(2.093–3.252)	0.000	7.532(5.284–10.736)	0.000	3.738(2.465–5.669)
**HCV(for HIV and Syphilis)/Syphilis (for HCV)**												
Negative		Ref.		Ref.		Ref.		Ref.		Ref.		Ref.
Positive	0.000	7.532(5.284–10.736)	0.000	4.117(2.702–6.274)	0.000	5.514(4.533–6.707)	0.000	4.357(3.463–5.481)	0.000	5.514(4.533–6.707)	0.000	4.157(3.315–5.213)

Note: FSW = Female Sex Worker;

*Hosmer-Lemeshow goodness-of-fit for HIV multivariate analysis model is p = 0.216;

¶Hosmer-Lemeshow goodness-of-fit for syphilis multivariate analysis is p = 0.039;

#Hosmer-Lemeshow goodness-of-fit for HCV multivariate analysis is p = 0.148.

**Table 5:** Multivariate models of correlates of the outcomes of testing positive for HIV, HCV, and syphilis among female sex workers (FSWs) in Guangxi Province, China, China National CDC HIV/AIDS Surveillance Data, 2010–2012,N = 51,790.

HIV Positive	AOR (CI)	Syphilis Positive	AOR (CI)	HCV Positive	AOR (CI)
**HCV positive**	4.12 (2.70–6.27)	**HCV positive**	4.36 (3.46–5.48)	**Ever Inject Drugs**	60.65 (43.42–84.72)
**Elementary School or less**	3.71(2.14–6.43)	**Prior STI in last 12 months**	3.56 (3.15–4.02)	**Syphilis Positive**	4.16 (3.32–5.21)
**Junior High School**	2.08 (1.20–3.60)	**HIV Positive**	2.61 (2.09–3.25)	**HIV Positive**	3.74 (2.47–5.67)
**Syphilis Positive**	2.56(2.06–3.20)	**Elementary School or less**	2.35 (1.97–2.80)	**Low Tier**	1.56 (1.06–2.29)
**Ever Inject Drugs**	2.10(1.04–4.23)	**Low Tier**	2.00 (1.69–2.37)	**Years in FSW > = 1**	1.32 (1.06–1.65)
**Low Tier**	2.02 (1.29–3.18)	**Age > = 35**	1.71 (1.56–1.86)		
**Divorced/Widowed**	1.82(1.24–2.67)	**Junior High School**	1.57 (1.32–1.87)		
**Age > = 35**	1.65(1.34–2.05)	**Divorced/Widowed**	1.33 (1.10–1.59)		
**Inconsistent Condom Use**	1.48(1.23–1.78)	**Not married/Single**	1.28 (1.16–1.41)		
**Prior STI in last 12 months**	1.40(1.02–1.94)	**Years in FSW > = 1**	1.19 (1.09–1.30)		
		**Not Guangxi**	1.11(1.02–1.21)		
		**Inconsistent Condom Use**	1.10 (1.01–1.18)		

Screening positive for syphilis was significantly associated with screening HCV positive (AOR 4.36; CI 3.46–5.48), STI in prior 12 months (AOR 3.56; CI 3.15–4.02), screening HIV positive (AOR 2.61; CI 2.09–3.25), only competing elementary school (AOR 2.35; CI 1.97–2.80) or junior high school (AOR 1.57; CI1.32–1.87), being at low tier venue (AOR 2.00; CI 1.69–2.37), being divorced/widowed or single (AOR 1.33; CI 1.10–1.59 and AOR 1.28; CI 1.16–1.41), age greater or equal to 35 (AOR 1.71; CI 1.56–1.86), and working more than one year in sex work in the region (AOR 1.19; CI 1.09–1.30). Interestingly, inconsistent condom use only had an AOR of 1.10 (CI 1.01–1.18) of increased odds for syphilis acquisition (Table 4,Demographic and risk factors correlations of HIV or syphilis infection among female sex workers (FSW), Guangxi province, China, China National CDC HIV/AIDS Surveillance Data, 2010–2012).

HCV positivity was most correlated with ever injection drug use (AOR 60.65;CI 43.42–84.72), being syphilis positive (AOR 4.16;CI 3.32–5.21), and co-infection with HIV (AOR 3.74;CI 2.47–5.67) (Table 5, Multivariate models of correlates of the outcomes of testing positive for HIV, HCV, and syphilis among female sex workers (FSWs) in Guangxi Province, China, China National CDC HIV/AIDS Surveillance Data, 2010–2012, N = 51,790).

## Discussion

This comprehensive, coordinated behavioral survey and screening campaign of 51,790 FSWs in Guangxi Province under the auspices of the China CDC National HIV/AIDS Sentinel Surveillance project represents one of the largest combined survey and laboratory screening projects for FSWs in southern China.

This study demonstrates the success of the HIV/AIDS harm reduction condom distribution program, outreach interventions, regular HIV screening (every 6 months) supported by multilevel government departments in China and the Global Fund in reducing HIV risk behavior among Chinese FSWs [38–39]. Condom distribution, outreach intervention and regular HIV screening with evolution to safer sex practices are likely the reasons to explain the non-increasing prevalence of HIV, HCV, and syphilis in Guangxi for 2010–2012. Even though a different sample of FSW were included each year, making it difficult to link an individual’s consistent condom use with testing outcomes, but the results are valid on a population level and demonstrate similar to previous studies that low tier FSW utilized condoms least frequently in comparison to other types of FSWs [20,28,40].

Our data also show a very high prevalence of ever injection drug use among low tier FSWs that is not changing over the study period. Ever injection drug use for low tier FSWs in Guangxi was found to be extremely high at 30.3% among those who ever used drugs, which rivals the high prevalence of Yunnan province low tier FSWs [18], who could be part of the most high risk transmission bridging group. With other factors held constant, women with severe drug dependency are more likely than men to be at increased risk of sexual acquisition of HIV due to greater biological and social vulnerability [41]. Acquiring drugs requires money or access to resources, and most female drug users in China trade sex for money or drugs [42]. Overall, women intravenous drug users are more likely than their male counterparts to engage in high-risk sex with multiple partners for money or drugs, share needles, and have unprotected sex with an IDU partner[43].

HCV infection among people who acquired HIV-1 infection through sexual contacts in 6 provinces in China was 20.1%[44], the HCV prevalence for general population, such as voluntary blood donors from large cities in China such as Beijing, Chongqing, and Jinan has been reported to be <1% [45–47]. Among outpatients and inpatients in a Liaoning provincial hospital and people who underwent routing health examination, only 0.55% were found to be HCV positive [45]. In our study, only low tier FSW showed HCV prevalence higher than 1%, other tiers still kept under 1% and stable, which meant Guangxi HCV prevalence through commercial sexual contacts is still low. As HCV positive FSWs were also at higher risk for testing positive for HIV and syphilis, interventions among FSWs to decrease intravenous drug use will likely further decrease HIV acquisition and transmission to the general population.

As the injection drug prevalence in Guangxi among all groups of FSWs remained constant over 2010–2012 and was clearly the highest risk factor for HCV acquisition and highly correlated to HIV transmission, we would suggest to expand current methadone maintenance treatment (MMT) and counseling centers that were started in 2004. Though 71 established MMT clinics in Guangxi cover 63 cities/counties (70% of the province) with active clients, further attention needs to be paid around FSW venues as to stem the tide of ongoing HIV infection that bridges high risk FSWs to the general population.

This study confirms that low tier FSWs are an extremely vulnerable risk group that warrants more prevention and risk reduction strategies. This group tends to have less formal education, less awareness of HIV/AIDS prevention, and be older in age (n age n, less awand thus have limited employment opportunities, receive low pay for other service jobs [28,48], and receive lower sex transaction payments (ex CNY / US $8 per transaction). As many support children of their own, this group is often more likely to engage in unprotected commercial sex with clients [49]. Clients of low tier FSW heterosexual clients tend to be men of equally low socio-economic status who also may be widowed, retired, or married to invalid or frail spouses and thus tend to seek paid sexual experiences outside of marriage [17,28] In Guangxi, elderly man were more likely than younger men to have commercial sex with low tier FSWs [50], since older clients often did not want to use condoms due to age related erectile dysfunction [17].As the HIV prevalence of these older male clients has already approached that of low tier FSWs [49,51], China is now witnessing a greater proportion of de novo HIV infections among elderly males [19,49,52,53]. As total HIV prevalence of FSWs in Guangxi province was shown in this surveillance study to be 0.92–1.10%, which is higher than that of other provinces in China except for 10.3% seen in Kaiyuan city of Yunnan Province [54], more attention should be paid to developing targeted interventions to low tier FSWs and their older male clients. Since only syphilis prevalence increased significantly among low tier FSWs between 2010–2012, it can be explained that, syphilis transmission through unprotected sex is higher than HIV in this mainly heterosexual population. Also, a higher syphilis positive percentage may have been explained by serofast status, in which a person may test positive due to past infection. For example, a German study demonstrated that newly HIV diagnosed patients have syphilis sero-prevalence of 20.3%, but only 6.7% with active syphilis (VDRL≥1:8) [55]. As HIV transmission has been shown to be more efficient in the presence of active syphilis infection as well as STI acquisition is a marker for high risk unprotected sexual activity, it is very important to create a dialogue to address STI in the efforts to reduce HIV[56]. Anonymous and free screening for HIV and other STD and the inter-disciplinary health education centers are strongly recommended to HIV/AIDS prevention and control strategy [57].

Limitations of this study include the enormous volume of questionnaires administered that may include varying quality and completeness of data due to chaotic environments or clients declining to answer. However, we note that most surveys had adequate completed data for analysis, and we were able to find statistical and clinical significance due to the extremely large sample size. Second, FSWs who screened positive for HIV in the first two years of the study may not have participated in subsequent years of the study that may have resulted in an underestimation of total disease prevalence; thus, correlations between condom use and disease acquisition could only be analyzed on a population level. Third, though low tier FSWs were known to be higher risk for disease acquisition, it was not until 2012 that larger numbers of low tier FSWs were recruited; thus participation rate went from 31.5% in 2010 to 46.8% in 2012, which may have upwardly biased overall prevalence sampling and oversampled those with higher risk behaviors such as injection drug use. However, as the populations most at risk were surveyed, this helps more clearly define the most important drivers of HIV, HCV, and syphilis transmission in Guangxi.

## Conclusions

This China CDC National HIV/AIDS Sentinel Surveillance project, one of the largest combined survey and laboratory screening projects for FSWs in southern China, demonstrates the success of condom distribution and regular HIV screening and outreach intervention campaigns to stabilize the transmission of HIV, HCV, and syphilis among high risk FSWs groups. However, more harm reduction and educational interventions are needed among low tier FSWs, FSWs who inject drugs, and older male clients [40, 53, 58–59].

## References

[1] China Ministry of Health, UNAIDS WHO, National Center for AIDS/STD Control and Prevention, China CDC. (2011) Report of epidemic situation of AIDS in China, 2011.

[2] Qian H, Vermund SH, Wang N (2005) Risk of HIV/AIDS in China: subpopulations of special importance. Sex Transm Infect 81(6): 442–447. 16326842 10.1136/sti.2004.014258 PMC1745065

[3] Bulletin of Guangxi 2010 Sixth National Census Data. Nanning, China, 2011 (Mandarin Chinese)

[4] Chu TX, Levy JA (2005). Injection drug use and HIV/AIDS transmission in China. Cell Res. Nov-Dec 2005;15(11–12):865–869. 16354561 10.1038/sj.cr.7290360

[5] Yu XF, Chen J, Shao Y, Beyrer C, Lai S (1998) Two subtypes of HIV-1 among injection-drug users in southern China. Lancet 351: 1250 9643749 10.1016/S0140-6736(05)79316-8

[6] Yu XF, Chen J, Shao Y, Beyrer C, Liu B, et al (1999) Emerging HIV infections with distinct subtypes of HIV-1 infection among injection drug users from geographically separate locations in Guangxi Province, China. J Acquir Immune Defic Syndr 22: 180–188. 10843533 10.1097/00126334-199910010-00011

[7] Su L, Graf M, Zhang Y, Von Briesen H, Xing H, et al (2000) Characterization of a virtually full-length human immunodeficiency virus type 1 genome of a prevalent intersubtype (C/B’) recombinant strain in China. J Virol 74:11367–11376. 11070037 10.1128/jvi.74.23.11367-11376.2000 PMC113242

[8] Piyasirisilp S, McCutchan FE, Carr JK, Sanders-Buell E, Liu W, et al (2000) A recent outbreak of human immunodeficiency virus type 1 infection in southern China was initiated by two highly homogeneous, geographically separated strains, circulating recombinant form AE and a novel BC recombinant. J Virol 74: 11286–11295. 11070028 10.1128/jvi.74.23.11286-11295.2000 PMC113233

[9] Beyrer C, Razak MH, Lisam K, Chen J, Lui W, et al (2000) Overland heroin trafficking routes and HIV-1 spread in south and south-east Asia. Aids 14:75–83. 10714570 10.1097/00002030-200001070-00009

[10] Tee KK, Pybus OG, Li XJ, Han X, Shang H, et al (2008) Temporal and spatial dynamics of human immunodeficiency virus type 1 circulating recombinant forms 08_BC and 07_BC in Asia. J Virol 82: 9206–9215. 10.1128/JVI.00399-08 18596096 PMC2546895

[11] Li L, Chen L, Liang S, Liu W, Li T. et al (2013)Subtype CRF01_AE dominate the sexually transmitted human immunodeficiency virus type 1 epidemic in Guangxi, China. J Med Virol. 85(3):388–395. 10.1002/jmv.23360 23341369

[12] Qi H, Zhao K, Xu F, Zhang X, Zhang Z. et al(2013) HIV-1 diversity, drug-resistant mutations, and viral evolution among high-risk individuals in phase II HIV vaccine trial sites in southern China. PLoS One.8(7):e68656 10.1371/journal.pone.0068656 23869225 PMC3711821

[13] Wang N, Wei H, Xiong R, Zhang H, Hsi JH, et al (2014)Near full-length genome characterization of a new CRF01_AE/CRF08_BC recombinant transmitted between a heterosexual couple in Guangxi, China. AIDS Res Hum Retroviruses. 30(5):484–8. 10.1089/AID.2013.0230 24164454 PMC4010168

[14] Zhao K, Du J, Zheng W, Yu XF. (2014) HIV-1 transmission among injection drug users leads to novel recombinants circulating in southern China.AIDS Res Hum Retroviruses. 10.1089/AID.2014.0036 PMC4180304 25103676

[15] Yang X, Abdullah AS, Wei B, Jiang J, Deng W, et al (2012) Factors influencing Chinese male’s willingness to undergo circumcision: A cross-sectional study in western China. PLoS ONE 7(1): e30198 10.1371/journal.pone.0030198 22253919 PMC3257276

[16] Zhu QY, Tang ZZ (2013) HIV/AIDS transmission between spouses and intervention strategy for them. Zhejiang Prev Med11,25(11):31–36. (Mandarin Chinese)

[17] Choi SY, Holroyd E. (2007) The influence of power, poverty and agency in the negotiation of condom use for female sex workers in mainland China. Cul Health Sex 9(5):489–503. 17687674 10.1080/13691050701220446

[18] Wang HB, Chen RY, Ding GW, Ma Y, Ma J, et al (2009) Prevalence and predictors of HIV infection among female sex workers in Kaiyuan City, Yunnan Province, China. Int J Infect Dis 13(2):162–169. 10.1016/j.ijid.2008.05.1229 18718801 PMC2650737

[19] Wang Y, Tang ZZ, Zhu QY, Liu W, Zhu JH, et al (2013) Epidemiological characteristics of HIV/AIDS in Guangxi, 2009–2011. South China J Prev Med 39(1):6–11. (Mandarin Chinese)

[20] Li J, Chen XS, Merli MG, Weir SS, Henderson GE. (2012) Systematic differences in risk behaviours and syphilis prevalence across types of female sex workers: a preliminary study in Liuzhou, China. Sex Transm Dis 39 (3):195–200. 10.1097/OLQ.0b013e31823d2e2a 22337106 PMC3282016

[21] National Center for AIDS/STD Control, Chinese Centers for Diseases Control and Prevention. National AIDS Sentinel Surveillance Guidelines. (2009) 3, 2009 (Mandarin Chinese)

[22] National Center for AIDS/STD Control, Chinese Centers for Diseases Control and Prevention. National AIDS Sentinel Surveillance Guidelines. (2010) 3 2010 (Mandarin Chinese)

[23] Chen XS, Liang GJ, Wang QQ, Yin YP, Jiang N, et al (2012) HIV prevalence varies between female sex workers from different types of venues in Southern China. Sex Transm Dis 39(11):868–870. 10.1097/OLQ.0b013e318264c3ba 23064536

[24] Yang Z, Su J, Peng X, Wu N (2013) A decline in HIV and syphilis epidemics in Chinese female sex workers (2000–2011): A systematic review and meta-analysis. PLoS ONE 8(12): e82451 10.1371/journal.pone.0082451 24349288 PMC3862622

[25] Wang L, Li DM, Ge L, Ding ZW, Wang L, et al(2013) HCV prevalence among the populations under the HIV sentinel surveillance data from 2009 to 2012 inChina. Zhonghua Liu Xing Bing Xue Za Zhi,34(6):543–547. (Mandarin Chinese) 24125599

[26] Li D, Wang L, Lin W, Li P, Wang L, et al (2014) HIV and syphilis infections among street-based female sex workers in China, 2010–2012. Chin Med J.127(4):707–711. 24534227

[27] Chen XS, Wang QQ, Yin YP, Liang GJ, Jiang N, et al (2012) Prevalence of syphilis infection in different tiers of female sex workers in China: implications for surveillance and interventions. BMC Infect Dis 12:84 10.1186/1471-2334-12-84 22475187 PMC3352076

[28] Zhou YJ, Tang ZZ, Shen ZY, Zhang XJ, Tang GJ, et al (2013) Survey of HIV/AIDS knowledge and behaviors for low tier female sex workers in Guangxi. Chin J Public Health 29(4):579–581. (Mandarin Chinese)

[29] He YX, Ruan YH, Teng T, Hao QN, Qin GM, et al Community-based survey of drug use and sexual behavior among female injecting drug users. Chinese Journal of AIDS/STD 2003; 9:343–346. (Mandarin Chinese)

[30] Ruan Y, Cao XY, Qian HZ, Zhang L, Qin G, et al (2006) Syphilis among female sex workers in southwestern China: potential for HIV transmission. Sex Transmis 33(12):719–723. 16708055 10.1097/01.olq.0000218881.01437.99

[31] Lu F, Jia YJ, Sun XH, Wang L, Liu W, et al (2009) Prevalence of HIV infection and predictors for syphilis infection among female sex workers in southern China. Southeast Asian J Trop Med Public Health 40(2):263–272. 19323011 PMC2745968

[32] Ding YP, Detels R, Zhao ZW, Zhu Y, Zhu G, et al (2005) HIV infection and sexually transmitted diseases in female commercial sex workers in China. J Acquir Immune Defic Syndr 38(3):314–319. 15735451

[33] Yi H, Mantell JE, Wu R, Lu Z, Zeng J, et al (2010) A profile of HIV risk factors in the context of sex work environments among migrant female sex workers in Beijing, China. Psychol Health Med 15(2):172–187. 10.1080/13548501003623914 20391235 PMC2856119

[34] Xia GM, Yang XS. (2005) Risky sexual behaviour among female entertainment workers in China: implications for HIV/STD prevention intervention. AIDS Educ Prev 17(2):143–156. 15899752 10.1521/aeap.17.3.143.62904

[35] Choi SY, Holroyd E. (2007) The influence of power, poverty and agency in the negotiation of condom use for female sex workers in mainland China. Cul Health Sex 9(5):489–503. 17687674 10.1080/13691050701220446

[36] Buzdugan R, Halli SS, Cowan FM (2009) The female sex work typology in India in the context of HIV/AIDS. Trop Med Int Health 14(6):673–687. 10.1111/j.1365-3156.2009.02278.x 19392742

[37] Harcourt C, Donovan B. (2005) The many faces of sex work. Sex Trans Infect 81(3):201–206. 15923285 10.1136/sti.2004.012468 PMC1744977

[38] Zou HC, Xue H, Wang XF, Lu D. (2012) Condom use in China: prevalence, policies, issues and barriers. Sexual Health 9:27–33. 10.1071/SH11021 22348630

[39] Zhang L, Liang S, Lu W, Pan SW, Song B, et al (2014) HIV, syphilis, and behavioral risk factors among female sex workers before and after implementation of harm reduction programs in a high drug-using areas of China. PLoS ONE 9(1): e84950 10.1371/journal.pone.0084950 24416319 PMC3885653

[40] Wang H, Reilly KH, Brown K, Jin X, Xu J, et al (2012) HIV incidence and associated risk factors among female sex workers in a high HIV-prevalence area of China. Sex Transm Dis.39(11): 835–841. 10.1097/OLQ.0b013e318266b241 23064531

[41] Pinkham S, Malinowska-Sempruch K. (2008) Women, Harm Reduction and HIV. Reprod Health Matters. 16(31):168–181. 10.1016/S0968-8080(08)31345-7 18513618

[42] Ming ZQ, Liang SL, Lorraine Y, Liu W, Wu ZY. (2002)Qualitative study of drug using and sexual behaviors of drug users in Guangxi. Chin J Epidemiol 23(11):1–3. 12015092

[43] Syvertsen JL, Robertson AM, Strathdee SA, Martinez G, Rangel MG, et al(2014) Rethinking risk: Gender and injection drug-related HIV risk among female sex workers and their non-commercial partners along the Mexico-U.S. border. Int J Drug Policy. 25(5):836–844. 10.1016/j.drugpo.2014.02.005 24641906 PMC4133300

[44] Shang H, Zhong P, Liu J, Han X, Dai D, et al (2010) High Prevalence and Genetic Diversity of HCV among HIV-1 Infected People from Various High-Risk Groups in China. PLoS ONE 5(5): e10631 10.1371/journal.pone.0010631 20523729 PMC2877711

[45] Wang Y, Tao QM, Zhao HY, Tsuda F, Nagayama R, et al (1994) Hepatitis C virus RNA and antibodies among blood donors in Beijing. J Hepatol 21: 634–640. 7529274 10.1016/s0168-8278(94)80112-6

[46] Zhao SM, Jiang TL, Li RQ, Gao FX, Lu L, et al (2008) HCV infection in voluntary donors and its influence on recruitment of donors in Chongqing area. Zhongguo Shi Yan Xue Ye Xue Za Zhi 16: 676–680. 18549653

[47] Ma X CY, Fu J, Chu Z (2007) Analysis of hepatitis C virus among 163854 blood donors in Jinan. Chinese Journal of Epidemiology 28: 733.

[48] Hao C, Liu HJ, Sherman SG, Jiang B, Li X, et al (2014) Typology of older female sex workers and sexual risk for HIV infection in China: a qualitative study.CulHealth Sex 16(1): 47–60. 10.1080/13691058.2013.826820 23998493 PMC4010200

[49] Liu HJ, Lin XQ, Xu YF, Chen SH, Shi J, et al(2012) Emerging HIV epidemic among older adults in Nanning, China. AIDS Patient Care STDs 26(10):565–567. 10.1089/apc.2012.0227 22984779 PMC3462385

[50] Chen Y, Tang ZZ, Shen ZY, Lan GH, Zhang HM, et al (2013) Investigation on the risk factors for HIV infection among elderly male clients of the commercial female sex-workers in Guangxi Zhuang Autonomous Region, in 2012. Chin J Epidemiol 34(11):1086–1090. (Mandarin Chinese) 24517940

[51] Buzdugan R, Halli SS, Cowan FM (2009) The female sex work typology in India in the context of HIV/AIDS. Trop Med Int Health 14(6):673–687. 10.1111/j.1365-3156.2009.02278.x 19392742

[52] Li J, Chen X, Qin BY. (2010) Investigation of HIV-related risk factors among elderly HIV-positive patients, Prac Prev Med 2(17): 227–229. (Mandarin Chinese)

[53] Hu XY, Qin SL, Chen X. (2010) Influence of social networks on the behaviorof sex workers. J Pub Health and Prev Med 21(5):45–47 (Mandarin Chinese).

[54] Wang HB, Chen RY, Sharp GB, Brown K, Smith K, et al(2010) Mobility, risk behavior and HIV/STI rates amongfemale sex workers in Kaiyuan City, Yunnan Province, China. BMC Inf Dis 10:198 10.1186/1471-2334-10-198 20615260 PMC2914052

[55] Spornraft-Ragaller P, Schmitt J, Stephan V, Boashie U, Beissert S.(2014)Characteristics and coinfection with syphilis in newly HIV-infected patients at the University Hospital Dresden 1987–2012. J Dtsch Dermatol Ges. 12(8):707–16. 10.1111/ddg.12382 24941863

[56] US-CDC.HIV prevention through early detection and treatment of other sexually transmitted diseases—United States. Recommendations of the Advisory Committee for HIV and STD prevention. (1998)MMWR Recomm Rep. 47(RR-12):1–24. 9701544

[57] Deniaud F, Melman C. (2002)From the apprehension of sexually transmissible diseases to the prevention of HIV. Presse Med. 31(9):387–392. 11933733

[58] Tran BX, Nguyen TV, Pham QD, Nguyen PD, Khuu NV, et al (2014) HIV infection, risk factors, and preventive services utilization among female sex workers in the Mekong Delta Region of Vietnam. PLoS ONE 9(1): e86267 10.1371/journal.pone.0086267 24475096 PMC3901683

[59] Wen XQ (2013) An analysis on knowledge, condom use and risk behavior related to HIV/AIDS in low tier female sex workers in Guilin. Mod Prev Med 40(15):2848–2855.

